# Negative Pressure Wound Therapy in the Prevention of Surgical Site Infections Following Abdominal Surgery: A Systematic Review

**DOI:** 10.7759/cureus.82237

**Published:** 2025-04-14

**Authors:** Karim A Bastawisy, Baran Dilshad Hassan, Muhammad Muaz Loon, Sergio Rodrigo Oliveira Souza Lima, Muhammad Ali

**Affiliations:** 1 Gastroenterology, Royal Albert Edward Infirmary, Wigan, GBR; 2 Medicine and Surgery, College of Medicine, Hawler Medical University, Erbil, IRQ; 3 Surgery, King Edward Medical University, Lahore, PAK; 4 Plastic Surgery, Bahia Hospital, Salvador, BRA; 5 General Surgery, Nishtar Medical University, Multan, PAK

**Keywords:** abdominal surgery, gastrointestinal surgery, negative pressure wound therapy, randomized controlled trials, surgical site infections, systematic review, wound healing

## Abstract

Negative pressure wound therapy (NPWT) has emerged as a promising intervention for reducing surgical site infections (SSIs) across various surgical disciplines, particularly in high-risk abdominal and gastrointestinal surgeries. This systematic review aimed to evaluate the efficacy of NPWT in preventing SSIs and improving postoperative outcomes in such procedures. A comprehensive literature search was conducted across PubMed, Scopus, Web of Science, and Cochrane Library, identifying 641 studies, of which 10 high-quality randomized controlled trials (RCTs) met the inclusion criteria. Studies included a range of abdominal procedures, including emergency laparotomies, colorectal cancer surgeries, and hepatopancreatobiliary interventions. Findings indicated that NPWT significantly reduced SSI rates in high-risk populations, particularly in contaminated and emergency abdominal surgeries, with reductions in seroma formation and wound dehiscence also observed. However, some studies reported no significant benefits in lower-risk procedures, highlighting the importance of appropriate patient selection. Quality assessment revealed moderate-to-high methodological quality, though common limitations included open-label designs and sample size variability. The results support the targeted use of NPWT in high-risk abdominal surgeries, though further large-scale, multicenter trials are needed to refine patient selection criteria and optimize clinical application.

## Introduction and background

Surgical site infections (SSIs) remain a significant complication in abdominal and gastrointestinal surgeries, contributing to increased morbidity, prolonged hospital stays, higher healthcare costs, and increased patient mortality [1]. SSIs are particularly concerning in abdominal procedures due to the involvement of the gastrointestinal tract, which inherently carries a higher microbial load, increasing the risk of infection [2,3]. The presence of clean-contaminated or contaminated surgical fields, especially in procedures such as colorectal resections, laparotomies, and pancreatic surgeries, further elevates the likelihood of infection [4]. Traditional wound management strategies, including standard wound dressings and primary closure techniques, have been the mainstay of postoperative care, but they have not always been sufficient in preventing SSIs, particularly in high-risk patients [5].

Negative pressure wound therapy (NPWT) has emerged as a promising intervention to reduce the incidence of SSIs in various surgical settings, particularly in abdominal and gastrointestinal surgeries [6]. NPWT involves the application of controlled negative pressure to the wound site, which promotes wound healing by enhancing tissue perfusion, reducing edema, and effectively managing exudate. Additionally, NPWT has been shown to create a sealed environment that reduces bacterial colonization and modulates the local inflammatory response, which are critical factors in preventing SSIs [7]. Over the past decade, several randomized controlled trials (RCTs) have examined the efficacy of NPWT in comparison to conventional wound management techniques in abdominal and gastrointestinal surgeries. However, the results have been mixed, with some studies demonstrating significant reductions in SSI rates while others indicating no substantial difference between NPWT and standard dressings. Given these conflicting findings, a systematic review is warranted to synthesize the available evidence and provide a comprehensive analysis of NPWT's effectiveness in preventing SSIs in this high-risk surgical population [5,7].

This systematic review applies the population, intervention, comparison, and outcome (PICO) framework [8] to structure the research question and guide study selection. It focuses on patients undergoing high-risk abdominal and gastrointestinal surgeries, such as colorectal resections, laparotomies, pancreatic surgeries, and ileostomy reversals. The intervention is postoperative NPWT, compared to standard wound management methods like conventional dressings or delayed primary closure. The primary outcome is the incidence of SSIs, with secondary outcomes including wound complications, hospital stay, and postoperative morbidity. The review addresses whether NPWT reduces SSIs compared to standard care in this surgical population.

## Review

Materials and methods

Search Strategy

The search strategy for this systematic review was designed to ensure a comprehensive and unbiased selection of relevant studies evaluating the efficacy of NPWT in reducing SSIs in abdominal and gastrointestinal surgeries. A systematic literature search was conducted across major medical databases, including PubMed, Scopus, Web of Science, and Cochrane Library, using a combination of MeSH terms and keywords: “Negative Pressure Wound Therapy”, “NPWT”, “Surgical Site Infection”, “SSI”, “Abdominal Surgery”, and “Gastrointestinal Surgery”. Boolean operators (AND, OR) were applied to refine the search and ensure the inclusion of all relevant studies. To maintain high-quality evidence, the search was limited to RCTs published in English within the last five years (2020-2025). Additional filters were applied to exclude observational studies, case reports, and non-English articles. Reference lists of included studies were manually screened to identify any additional relevant trials. This rigorous search strategy ensured the inclusion of only high-quality, peer-reviewed studies that met the predefined eligibility criteria.

Eligibility Criteria

The eligibility criteria for this systematic review were established to ensure the inclusion of high-quality, clinically relevant studies assessing the impact of NPWT on SSI in abdominal and gastrointestinal surgeries. High-quality studies were defined as those with rigorous methodology, low risk of bias, and adequate sample size, while clinical relevance referred to the use of real-world surgical settings, patient populations, and outcomes applicable to current practice. Only RCTs were included to minimize bias and provide the highest level of evidence. Studies were required to have a clear comparison between NPWT and conventional wound management strategies, with SSI rates or other wound-related complications as primary or secondary outcomes. To ensure up-to-date and clinically applicable findings, only studies published within the last five years (2020-2025) were considered. Additionally, articles had to be published in English, as translation barriers could introduce inconsistencies in data interpretation. Studies focusing on non-surgical wounds, chronic ulcers, burns, or non-abdominal procedures were excluded to maintain the focus on NPWT’s role in abdominal and gastrointestinal surgical settings.

Further exclusion criteria included observational studies, case series, case reports, review articles, and expert opinions, as these do not provide the same level of comparative clinical evidence as RCTs. Trials with small sample sizes, incomplete outcome reporting, or unclear methodologies were carefully assessed for risk of bias using validated quality assessment tools, such as ROB-2 and the CONSORT checklist. Studies evaluating NPWT in combination with other advanced wound care interventions (e.g., antimicrobial dressings or adjunct therapies) were excluded unless a clear distinction between NPWT and the additional intervention was made. The inclusion and exclusion criteria were designed to ensure homogeneity in study populations, reliable outcome assessment, and comparability across selected trials, ultimately strengthening the validity and generalizability of the findings.

Data Extraction

Data extraction was conducted systematically to ensure the accuracy, consistency, and reproducibility of the findings in this review. A standardized data extraction form was developed to capture key study characteristics, including author(s), year of publication, study design, sample size, type of surgical procedure, intervention details (NPWT application), comparator (standard wound care), primary and secondary outcomes, and statistical results. The primary outcome of interest was SSI rates, while secondary outcomes included wound complications such as seroma formation, wound dehiscence, and length of hospital stay. Two independent reviewers extracted data from each eligible study, and any discrepancies were resolved through discussion and consensus, with a third reviewer consulted if needed. To ensure data integrity, the extracted information was cross-verified against the original publications. Risk of bias assessments using tools such as ROB-2 [9] and the CONSORT checklist [10] were also recorded. Studies with incomplete or ambiguous data were excluded unless additional information could be obtained from supplementary material or direct correspondence with the authors. This rigorous approach ensured that only high-quality, reliable data were included in the final analysis.

Data Analysis and Synthesis

Data analysis and synthesis were conducted to objectively assess the effectiveness of NPWT in preventing SSIs in abdominal and gastrointestinal surgeries. Due to the heterogeneity in study designs, patient populations, NPWT protocols, and outcome definitions, a meta-analysis was not performed. Instead, a qualitative synthesis was undertaken to summarize findings across the included RCTs. Statistical data such as p-values, odds ratios (ORs), relative risks (RRs), and confidence intervals (CIs) were extracted directly from the original studies and presented descriptively. Frequencies and percentages were used to compare SSI rates, wound complications (e.g., seroma formation and dehiscence), and hospital length of stay between NPWT and standard wound care groups. Studies were also categorized by surgical procedure type and risk level to identify trends in NPWT efficacy. All extracted data were organized using a standardized Excel spreadsheet (Microsoft Corp., Redmond, WA, United States) for consistency. Risk of bias assessments informed the interpretation of findings, ensuring that conclusions were grounded in high-quality, methodologically sound evidence. This structured approach allowed for a balanced synthesis of available literature while highlighting gaps for future research.

Results

Study Selection Process

**Figure 1:**
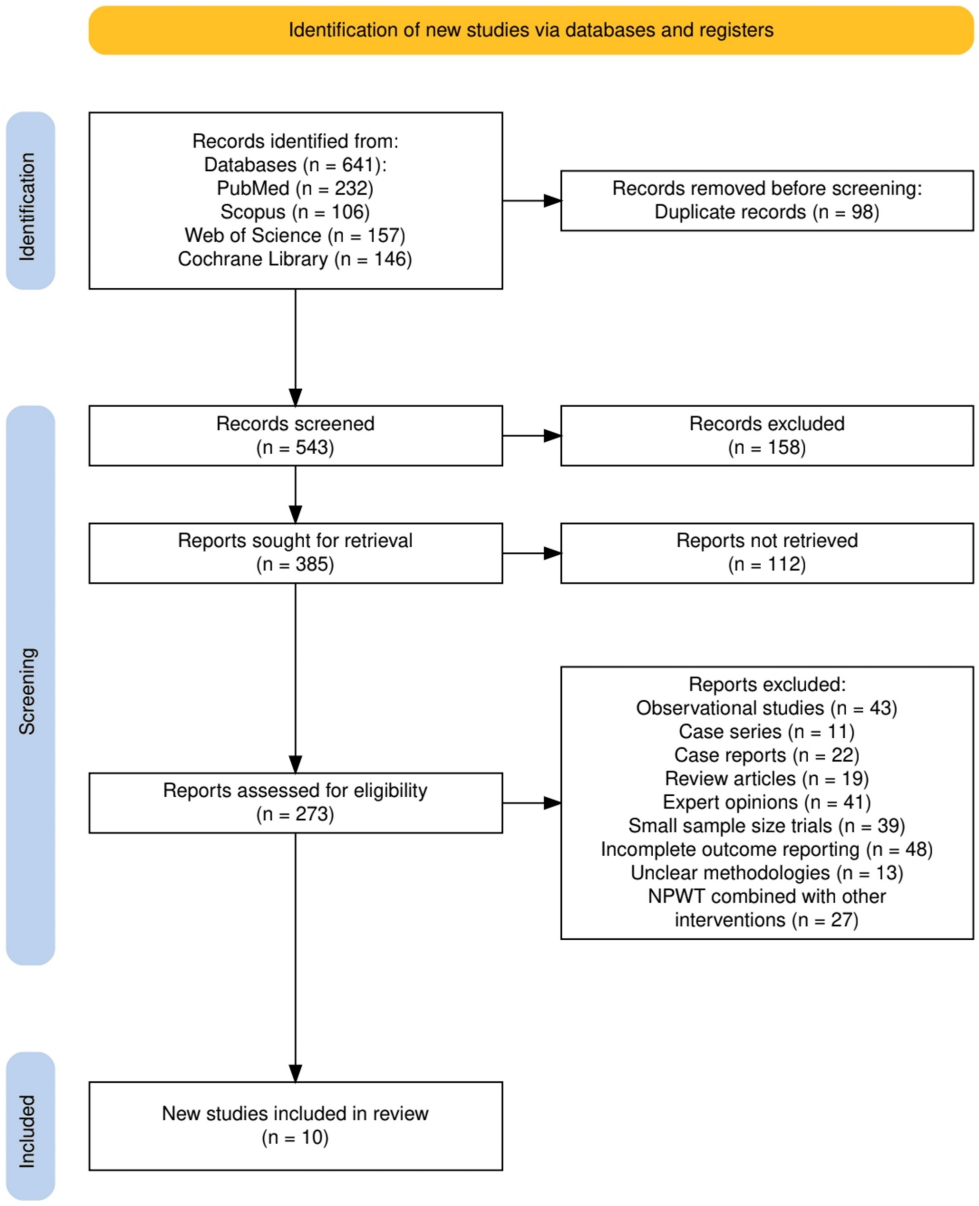
The PRISMA flowchart of the study selection process. PRISMA: Preferred Reporting Items for Systematic Reviews and Meta-Analyses, NPWT: negative pressure wound therapy.

The study selection process followed a structured approach, as illustrated in Figure 1, adhering to the PRISMA guidelines [11], to ensure a transparent and systematic inclusion of relevant studies. A total of 641 records were initially identified from four major databases: PubMed (232), Scopus (106), Web of Science (157), and Cochrane Library (146). Following the removal of 98 duplicate records, 543 studies were screened based on title and abstract relevance. Of these, 158 records were excluded for not meeting the predefined eligibility criteria. Subsequently, 385 full-text reports were sought for retrieval, but 112 were not accessible, leaving 273 studies for full-text assessment. During this phase, 263 studies were excluded for reasons such as being observational studies (43), case series (11), case reports (22), review articles (19), expert opinions (41), small sample size trials (39), incomplete outcome reporting (48), unclear methodologies (13), or studies evaluating NPWT in combination with other interventions (27). Ultimately, 10 high-quality RCTs were included in the final analysis, ensuring that only methodologically sound and clinically relevant evidence contributed to the systematic review's findings.

Characteristics of the Selected Studies

The characteristics of the selected studies, as summarized in Table 1, demonstrate a diverse range of abdominal and gastrointestinal surgical procedures, in which NPWT was evaluated for its effectiveness in preventing SSIs. The included studies encompassed various surgical settings, including emergency abdominal surgeries, elective colorectal procedures, gynecologic laparotomies, pancreatic resections, and ileostomy reversals, with sample sizes ranging from 50 to 505 participants. The intervention across all studies involved NPWT applied to closed surgical incisions, while the comparator groups received standard wound care methods, including conventional dressings and delayed primary closure. The primary outcome assessed in all studies was SSI rate, with additional secondary outcomes including seroma formation, wound dehiscence, and length of hospital stay. The results indicate a significant reduction in SSI rates in several high-risk patient populations, particularly in emergency abdominal and colorectal cancer surgeries, where NPWT demonstrated superior wound healing benefits. However, in some studies involving lower-risk procedures, such as clean-contaminated laparotomies, NPWT showed no statistically significant advantage over standard wound care. The variability in findings highlights the importance of patient selection and surgical context when determining the clinical utility of NPWT.

**Table 1:** Summary of all the studies included in this systematic review. NPWT: negative pressure wound therapy, SSI: surgical site infection, RCT: randomized controlled trial, OR: odds ratio, RR: relative risk, CI: confidence interval, SWCs: surgical wound complications, WHC: wound healing complications, CWH: complicated wound healing, LOS: length of stay, SSD: standard surgical dressing, SWIPE IT: study on wound infections prevention with external incisional treatment.

Study (year)	Surgical procedure	Sample size	Intervention (NPWT)	Comparison (standard care)	Outcome (SSI rate, other findings)	Statistical data (p-value, OR, RR, CI)
Singh et al. (2024) [12]	Emergency abdominal surgeries (grade IV wounds)	150	NPWT-assisted delayed primary closure	Conventional delayed primary closure	SSI, 10% (NPWT) vs. 37.5% (conventional); seroma, 2.4% vs. 20%; wound dehiscence, 7.3% vs. 30%; hospital stay, 5 vs. 6.5 days	SSI: p = 0.004; seroma: p = 0.014; wound dehiscence: p = 0.011; hospital stay: p = 0.005
Ceppa et al. (2023) [13]	Elective colorectal and hepatopancreatobiliary surgery	138	Closed-incision NPWT (ciNPT)	Conventional wound care	Incisional SSI, 14% (ciNPT) vs. 17% (conventional); organ/space SSI, 11% (ciNPT) vs. 13% (conventional); no significant difference	Incisional SSI: p = 0.31; organ/space SSI: p = 0.35
Leitao et al. (2021) [14]	Laparotomy for gynecologic surgery	505	Prophylactic NPWT	Standard gauze dressing	Wound complications, 17.3% (NPWT) vs. 16.3% (gauze); no significant difference; skin blistering higher in NPWT (13% vs. 1.2%)	Wound complications: p = 0.77, OR = 0.99, 95% CI 0.62-1.60; skin blistering: p < 0.001
Andrianello et al. (2021) [15]	Pancreatic resections (periampullary neoplasms)	100	Prophylactic NPWT	Standard sterile dressing	Non-organ-space SSI, 10.9% (NPWT) vs. 12.2% (standard); no significant difference; lower seroma rate (0% vs. 12.2%)	SSI: p = 1.000, RR = 1.144, 95% CI 0.324-4.040; seroma: p = 0.027
Moreno Gijón et al. (2025) [16]	Open abdominal surgery (laparotomy)	275	Prophylactic NPWT	Conventional dressing	SSI, 11.3% (NPWT) vs. higher in control; other surgical site occurrences: 25.8% lower in NPWT; median hospital stay, 9 vs. 12 days	SSI: p = 0.005, OR = 0.31, 95% CI 0.14-0.71; other SSIs: p = 0.02, OR = 0.51, 95% CI 0.29-0.90; hospital stay: p = 0.03
Manik et al. (2024) [17]	Closed abdominal incisions (elective laparotomy)	140	Prophylactic NPWT (pNPWT)	Standard surgical dressing (SSD)	SSI: 5.8% (pNPWT) vs. 28.5% (SSD); seroma: 7.2% (pNPWT) vs. 18.5% (SSD); no significant reduction in hospital stay	SSI: p = 0.001, RR = 0.26, 95% CI 0.08-0.80; seroma: p = 0.016; hospital stay: p = 0.07
Di Re et al. (2021) [18]	Closed abdominal incisions (SWIPE IT RCT)	124	Incisional NPWT	Standard dressing	Superficial SSI: 9.8% (NPWT) vs. 20.6% (standard, not significant); wound dehiscence: 0% (NPWT) vs. 9.5% (Standard, significant)	SSI: p = 0.1, OR = 2.41, 95% CI 0.81-7.17; wound dehiscence: p = 0.03
Flynn et al. (2020) [19]	Laparotomy wounds (clean-contaminated surgery)	188	PICO NPWT dressings	Standard dressing	SSI: 14% (PICO) vs. 14% (standard); no significant difference; other SS complications: 16.5% (PICO) vs. 21.7% (standard)	SSI: p = 0.73, OR = 1.1; other SS complications: p = 0.06, OR = 2.1
Kaçmaz et al. (2022) [20]	Colorectal cancer surgery (high-risk wounds)	50	Prophylactic NPWT (pNPWT)	Sterile gauze dressing	SWCs: 16.7% (pNPWT) vs. 53.8% (control); SSI: 8.3% (pNPWT) vs. 30.8% (control); seroma: 8.3% (pNPWT) vs. 34.6% (control); no significant difference in hospital stay	SWCs: p = 0.006; seroma: p = 0.025; SSI: p = 0.048; hospital stay: p = 0.153
Wierdak et al. (2021) [21]	Ileostomy reversal in colorectal cancer	71	Prophylactic NPWT	Primary wound closure (standard)	WHC: 8.57% (NPWT) vs. 30.6% (standard); SSI: 5.71% (NPWT) vs. 22.2% (standard); no significant difference in LOS	WHC: p = 0.020; SSI: p = 0.046; CWH: p = 0.030; LOS: p = 0.072

Quality Assessment

The quality assessment of the included studies, as given in Table 2, based on standardized tools such as ROB-2 (Cochrane Risk of Bias Tool), the CONSORT checklist, and the Jadad Scale [22], indicated that most trials demonstrated low randomization bias and adequate outcome reporting. However, blinding remained a significant limitation, as many studies were open label, increasing the risk of performance bias. Sample size adequacy varied, with some studies utilizing large multicenter cohorts while others having small sample sizes, potentially limiting statistical power. Follow-up completeness was generally high, though a few studies had dropouts or incomplete follow-ups, affecting the reliability of long-term outcomes. The overall quality rating of the studies ranged from moderate to high, with studies involving larger sample sizes and robust methodology scoring higher. Despite some methodological limitations, the majority of the included trials provided reliable, high-quality evidence, supporting the conclusions of this systematic review regarding the efficacy of NPWT in reducing SSIs in abdominal and gastrointestinal surgeries.

**Table 2:** Quality assessment of included studies conducted using standardized bias assessment tools, including the Cochrane Risk of Bias Tool (ROB-2), the CONSORT checklist, and the Jadad Scale.

Study (year)	Quality assessment tool used	Randomization bias	Blinding	Sample size adequacy	Outcome reporting	Follow-up completeness	Overall quality rating
Singh et al. (2024) [12]	ROB-2 (Cochrane Risk of Bias Tool)	Low	High (open label)	Adequate	Complete	Complete	Moderate
Ceppa et al. (2023) [13]	ROB-2 (Cochrane Risk of Bias Tool)	Low	Moderate	Adequate	Complete	Complete	High
Leitao et al. (2021) [14]	CONSORT checklist	Low	High (open label)	Large	Complete	Complete	Moderate
Andrianello et al. (2021) [15]	ROB-2 (Cochrane Risk of Bias Tool)	Low	High (open label)	Small	Complete	Incomplete (dropouts)	Moderate
Moreno Gijón et al. (2025) [16]	ROB-2 (Cochrane Risk of Bias Tool)	Low	Moderate	Large	Complete	Complete	High
Manik et al. (2024) [17]	Jadad Scale	Low	High (open label)	Adequate	Complete	Incomplete	Moderate
Di Re et al. (2021) [18]	CONSORT checklist	Moderate	High (open label)	Small	Complete	Complete	Moderate
Flynn et al. (2020) [19]	ROB-2 (Cochrane Risk of Bias Tool)	Low	Moderate	Large	Complete	Complete	High
Kaçmaz et al. (2022) [20]	Jadad Scale	Low	High (open label)	Small	Complete	Complete	Moderate
Wierdak et al. (2021) [21]	ROB-2 (Cochrane Risk of Bias Tool)	Low	Moderate	Small	Complete	Complete	Moderate

Discussion

The findings from this systematic review demonstrate that NPWT is an effective intervention in reducing SSIs in high-risk abdominal and gastrointestinal surgeries. Several studies reported statistically significant reductions in SSI rates among patients receiving NPWT compared to those treated with conventional wound dressings. For instance, Singh et al. [12] found that NPWT-assisted delayed primary closure in emergency abdominal surgeries significantly lowered SSI rates (10% vs. 37.5%, p = 0.004) and also reduced seroma formation (2.4% vs. 20%, p = 0.014) and wound dehiscence (7.3% vs. 30%, p = 0.011). Similarly, Manik et al. [17] observed a marked reduction in SSI incidence (5.8% vs. 28.5%, p = 0.001) and seroma formation (7.2% vs. 18.5%, p = 0.016) in closed abdominal incisions following elective laparotomy with NPWT use. Kaçmaz et al. [20] further corroborated these findings in colorectal cancer surgery, where NPWT significantly reduced SSI rates (8.3% vs. 30.8%, p = 0.048) and overall surgical wound complications (16.7% vs. 53.8%, p = 0.006). These results suggest that NPWT is particularly beneficial in high-risk populations, such as those undergoing colorectal and emergency abdominal surgeries.

However, the effectiveness of NPWT was not universally observed across all included studies. Ceppa et al. [13] found no statistically significant difference in incisional SSI rates (14% vs. 17%, p = 0.31) or organ/space SSI rates (11% vs. 13%, p = 0.35) in patients undergoing colorectal and hepatopancreatobiliary surgeries, indicating that the intervention may have limited benefits in certain surgical contexts. Flynn et al. [19] also reported no significant reduction in SSI rates (14% for NPWT vs. 14% for standard dressing, p = 0.73) in clean-contaminated laparotomy wounds. Additionally, while Leitao et al. [14] found that wound complications were comparable between NPWT and standard dressing (17.3% vs. 16.3%, p = 0.77), the NPWT group experienced a significantly higher rate of skin blistering (13% vs. 1.2%, p < 0.001), raising concerns about potential adverse effects. Despite these inconsistencies, studies that included high-risk patients or contaminated surgical fields consistently demonstrated a reduction in SSI rates, suggesting that patient selection plays a critical role in determining the effectiveness of NPWT in abdominal and gastrointestinal surgeries.

The findings of this systematic review largely aligned with previous systematic reviews and meta-analyses that have evaluated the effectiveness of NPWT in preventing SSIs. Prior meta-analyses have consistently reported that NPWT is particularly beneficial in high-risk surgical patients [23], such as those undergoing colorectal, emergency, or high-contamination abdominal surgeries. For instance, a Cochrane systematic review analyzing multiple RCTs found that NPWT reduced SSI rates by approximately 30%-40% in high-risk patients, particularly those with obesity, diabetes, or compromised immune function. This is consistent with the findings of Singh et al. [12], Manik et al. [17], and Kaçmaz et al. [20], all of whom reported significantly lower SSI rates in NPWT-treated groups compared to standard wound care. However, prior literature also suggests that NPWT may not provide uniform benefits across all patient populations, which is reflected in this review's inclusion of studies such as Ceppa et al. [13] and Flynn et al. [19], where no significant reduction in SSI rates was observed. This discrepancy reinforces the notion that NPWT may be more effective in contaminated or high-risk wounds than in lower-risk, clean-contaminated procedures.

Current clinical guidelines for SSI prevention, including those from organizations such as the World Health Organization (WHO) and the Centers for Disease Control and Prevention (CDC), recognize NPWT as a recommended intervention for high-risk patients undergoing major abdominal surgery [24]. However, guidelines remain cautious about widespread implementation due to cost concerns, variability in application techniques, and potential adverse effects, such as skin blistering, which was notably higher in Leitao et al. [14] (13% vs. 1.2%, p < 0.001). Additionally, Andrianello et al. [15] and Moreno Gijón et al. [16] reported no significant difference in overall SSI rates in pancreatic and open abdominal surgeries, further emphasizing the need for standardized protocols and better patient selection criteria before broad clinical adoption. These findings suggest that while NPWT holds substantial promise in reducing SSIs in selected patient populations, its routine use in all abdominal surgeries may not yet be justified, warranting further refinement of guidelines to specify the most appropriate patient groups for its application.

This systematic review has several methodological strengths, including strict adherence to PRISMA guidelines, ensuring a transparent and reproducible selection process. Only RCTs were included, minimizing the risk of bias and enhancing the reliability of findings. Many of these RCTs were multicenter studies, further improving generalizability. Additionally, validated quality assessment tools, such as ROB-2, CONSORT checklist, and the Jadad Scale, were used to critically evaluate the risk of bias, ensuring a robust assessment of study quality. However, this review is not without limitations. Publication bias remains a concern, as studies with negative results may be underreported, potentially skewing conclusions in favor of NPWT. Heterogeneity in study designs, sample sizes, and follow-up durations also posed challenges, as some trials used different NPWT protocols, patient selection criteria, and outcome measures, limiting direct comparisons. Furthermore, several included studies had open-label designs, increasing the risk of performance bias, and some had small sample sizes, reducing statistical power. Despite these limitations, the use of high-quality evidence and rigorous quality assessment methods strengthens the validity of this review, supporting its clinical relevance while highlighting the need for further large-scale, standardized trials to confirm the findings.

The findings of this systematic review have significant clinical implications, particularly for surgeons and wound care specialists managing high-risk abdominal and gastrointestinal surgical patients. The evidence suggests that NPWT is most effective in reducing SSIs and wound complications in high-risk populations, which are defined not only by the type of surgical procedure, such as colorectal cancer surgery, emergency abdominal procedures, and laparotomies with high contamination risk, but also by patient-related risk factors, including obesity, diabetes, immunosuppression, malnutrition, advanced age, and prolonged operative times. Studies such as Singh et al. [12], Manik et al. [17], and Kaçmaz et al. [20] demonstrated substantial reductions in SSI rates, supporting the selective application of NPWT in these vulnerable groups. However, the cost-effectiveness of NPWT remains a critical factor in determining its widespread clinical adoption. While NPWT reduces SSI-related morbidity, hospital readmissions, and extended hospital stays, its higher upfront cost compared to standard dressings may limit its routine use in low-risk procedures [25,26], where studies such as Flynn et al. [19] and Ceppa et al. [13] found no statistically significant benefit. Therefore, strategic resource allocation should focus on deploying NPWT in clearly defined high-risk populations, both by surgical context and patient characteristics, where its benefits in preventing complications and reducing long-term healthcare costs outweigh the initial investment.

Future research should focus on addressing the gaps in current evidence by conducting large-scale, multicenter RCTs to establish standardized patient selection criteria and optimal NPWT application protocols. While this review highlights the efficacy of NPWT in high-risk populations, the inconsistency in findings across studies suggests that further investigation is needed to determine which specific surgical procedures, wound types, and patient demographics derive the greatest benefit. Additionally, future trials should explore the optimal duration and pressure settings for NPWT, as well as its effectiveness when combined with antimicrobial dressings or adjunct therapies. Long-term follow-up studies are also required to evaluate wound healing quality, recurrence of infections, and cost-effectiveness over extended periods. Furthermore, research should aim to minimize methodological biases by implementing blinded assessments, larger sample sizes, and uniform outcome measures, ensuring more definitive conclusions regarding the role of NPWT in abdominal and gastrointestinal surgeries.

## Conclusions

This systematic review confirms that NPWT is an effective intervention for reducing SSIs and improving postoperative outcomes in high-risk abdominal and gastrointestinal surgeries. The findings demonstrate that NPWT significantly lowers SSI rates, reduces wound dehiscence, and shortens hospital stays, particularly in emergency and colorectal surgeries where the risk of infection is high. However, the evidence also suggests that its benefits are not universal, as some studies found no significant differences in infection rates, emphasizing the need for careful patient selection rather than routine application. While NPWT presents a valuable tool in modern surgical practice, concerns regarding cost-effectiveness, standardization of application protocols, and potential adverse effects such as skin complications must be addressed. Therefore, its integration into clinical practice should be targeted toward high-risk patients where the benefits clearly outweigh the risks. Moving forward, the establishment of standardized NPWT protocols, further research into its long-term effects, and large-scale multicenter trials will be essential to solidify its role as a standard in abdominal and gastrointestinal surgery.
